# Stoichiometric Determination of Nitrate Fate in Agricultural Ecosystems during Rainfall Events

**DOI:** 10.1371/journal.pone.0122484

**Published:** 2015-04-07

**Authors:** Zuxin Xu, Yiyao Wang, Huaizheng Li

**Affiliations:** Institute of Water Environment Rehabilitation, College of Environmental Science and Engineering, Tongji University, Shanghai, China; CAS, CHINA

## Abstract

Ecologists have found a close relationship between the concentrations of nitrate (NO_3_^-^) and dissolved organic carbon (DOC) in ecosystems. However, it is difficult to determine the NO_3_^-^ fate exactly because of the low coefficient in the constructed relationship. In the present paper, a negative power-function equation (*r* ^2^ = 0.87) was developed by using 411 NO_3_^-^ data points and DOC:NO_3_^-^ ratios from several agricultural ecosystems during different rainfall events. Our analysis of the stoichiometric method reveals several observations. First, the NO_3_^-^ concentration demonstrated the largest changes when the DOC:NO_3_^-^ ratio increased from 1 to 10. Second, the biodegradability of DOC was an important factor in controlling the NO_3_^-^ concentration of agricultural ecosystems. Third, sediment was important not only as a denitrification site, but also as a major source of DOC for the overlying water. Fourth, a high DOC concentration was able to maintain a low NO_3_^-^ concentration in the groundwater. In conclusion, this new stoichiometric method can be used for the accurate estimation and analysis of NO_3_^-^ concentrations in ecosystems.

## Introduction

Since the Industrial Revolution, the amount of reactive nitrogen (N) species in the natural environment has increased by an order of magnitude, owing to the use of artificial fertilizers and fossil fuels [1]. This environment has led to an increase in food production [2]; however, up to 80% of the applied N in fertilizers may be transported by precipitation to the groundwater and surface water, with most N being in the form of nitrate (NO_3_^-^) [3,4]. These conditions increase the risk of ingesting drinking water that has been contaminated with nitrates, which can lead to methemoglobinemia (“blue baby syndrome”) [5] or stomach cancer in humans [6], NO_3_^-^ poisoning in animals [7], and eutrophication of aquatic ecosystems [8]. NO_3_^-^ is a product of nitrification and a reactant in denitrification processes [9,10]. Therefore, the fate of NO_3_^-^ has been a popular focus of examinations of the N cycle in the global biosphere [11,12].

Redfield [13] observed that planktonic biomass contains carbon (C), N, and phosphorus (P) in an atomic ratio of 106:16:1 (the “Redfield ratio”). This observation has prompted ecologists to search for relationships through the stoichiometric method [14]. The Redfield ratio is an efficient tool for estimating the nutrient balance of ecosystems [15]. Dissolved organic carbon (DOC) and NO_3_^-^ are important forms of C and N in streams [16]. Aitkenhead and McDowell [17] showed that the soil C/N ratio can influence riverine DOC flux, and that the DOC and NO_3_^-^ concentrations in streams are closely related [16].

Many scientists [16,18] have tried to determine NO_3_^-^ concentrations by examining changes in the DOC concentration in ecosystems. Taylor and Townsend [19] found that NO_3_^-^ exhibits a consistently negative and nonlinear correlation with organic carbon (OC) along a hydrologic continuum, from soils through freshwater systems and coastal margins to the open ocean. However, very low correlation coefficients (mean *r* ^2^ = 0.36) were obtained for the exponential equations.

Here, we report a field study investigating the NO_3_^-^ and DOC concentrations in agricultural ecosystems during rainfall events. Our goal was to determine NO_3_^-^ fate by using a new stoichiometric method.

## Materials and Methods

### Site Descriptions and Experimental Design

Permissions for farmland use were provided by Prof. Zhongxian Lin, who is the agricultural manager of the study sites on Chongming Island. For all other locations, permissions were provided by Jie Huang, who is vice-director of Chongming Island environmental management.

**Fig 1:**
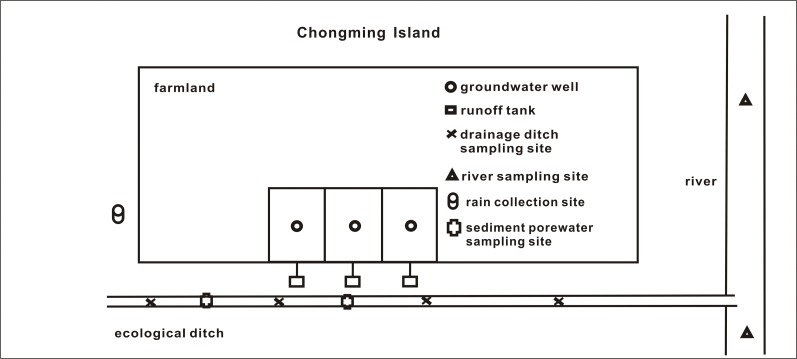
Study site and sampling locations.

Chongming Island (Fig 1) (30° 92′ N, 103° 62′ E) has a relatively flat topology and a subtropical monsoon climate. The average temperature is 15.7°C and average annual rainfall is 1100 mm, with 59.7% of rain falling between April and August. Most of the area of the island supports agricultural use. There are many rivers that are located near farmland, and the groundwater depth is below 1 m in most areas.

The study area contained three runoff tanks, three groundwater wells (~4 m in depth, 6 cm in diameter), four drainage ditches, two rivers, one rainfall collection site, and two sediment porewater sampling sites (Fig 1). Shallow ridges (20 cm in depth, 1% slope) around farmland were used to drain runoff to drainage ditches. Three runoff tanks were placed to collect runoff for this study. One drainage ditch (150 m × 4 m × 1.5 m, not hardened with cement), located in the western portion of the farmland area, was divided into four sections to support cane shoots, *Hydrilla*, and duckweed, with one unplanted control section. There were two river sites: a small site (11 m in width) located in the western 500 m of farmland, and a large site (20 m in width) located in the southern 1300 m of farmland. The rainfall collection site was installed on the western side of the farmland. The two sediment porewater sampling sites were located in a cane shoot ditch and an unplanted control ditch. The farmland was planted with vegetables and fertilized with swine litter and pig slurry.

### Sampling and Nutrient Measurement

We sampled different ecosystems (Fig 1) on Chongming Island from April to September of 2013: three farmland runoff systems, three farmland groundwater systems, four drainage ditch systems, two sites with sediment porewater, two rivers, one rainfall collection site, and seven indoor experimental ecosystems. Samples were collected during all rainfall events occurring in this period. All ecosystems were sampled simultaneously.

Sediment was sampled with a stainless gravity corer (40 cm in length and 5 cm in diameter). Sediment samples were sectioned in 5-cm layers (0–5, 5–10, 10–15, 15–20, and 20–25 cm). Large and small experimental ecosystems were composed of sediment, overlying water, plants (duckweed), and runoff in plastic boxes (S1 Table). Boxes were placed in an air-conditioned culture room to maintain the water temperature at about 25°C, with light supplied by electric bulbs. Runoff from artificial rainfall was added to the box to simulate NO_3_^-^ cycling (S1 Table). Details of the sampling process and weather are shown in (S2 Table). Samples were kept in a cool, dark environment until analysis (generally within 2 days). Porewater was obtained from the sediment by centrifuging the sediment mixture for 20 min at 4000 rotations/min (500 *g*) [20].

Duplicate samples were collected from all ecosystems to determine the DOC and NO_3_^-^ concentrations. All samples were filtered through a Millipore filter (pore size: 0.45 μm) into a 20-ml glass bottle. One filtered sample was treated with concentrated hydrochloric acid (pH < 2), and the DOC concentration was analyzed by a TOC5000A Total Organic Carbon Analyzer [16] (Shimadzu, Kyoto, Japan). The other filtered sample was analyzed by a standard colorimetric method to determine the NO_3_^-^ concentration [21].

### Data Analysis

**Fig 2:**
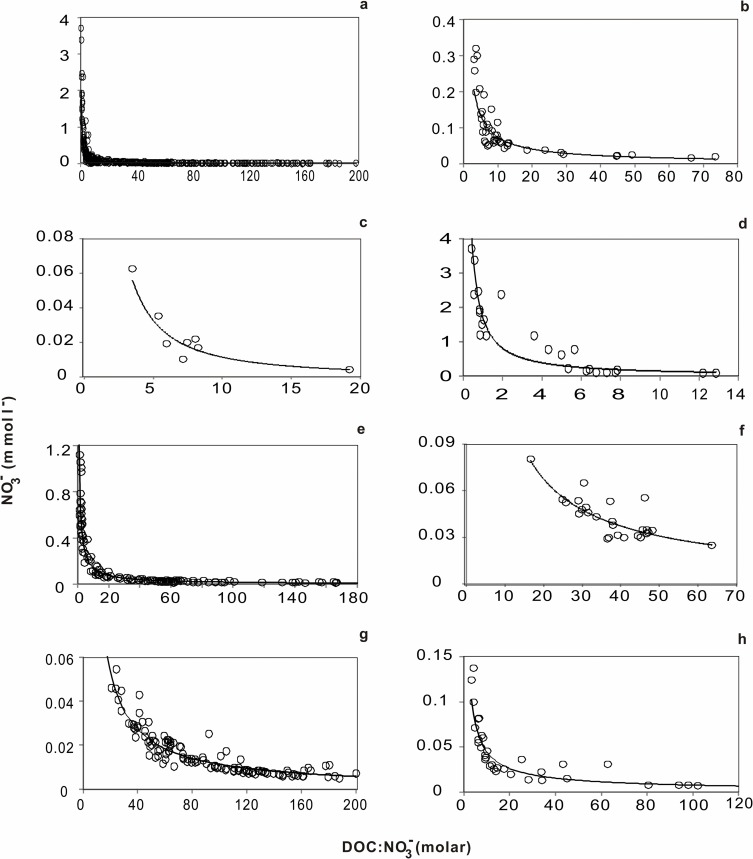
NO_3_- concentration as a function of changes in the molar DOC:NO_3_- ratio among major ecosystems of the rainfall transport route. a, all data; b, experimental systems; c, rainfall; d, runoff; e, drainage ditch; f, porewater; g, groundwater; h, river.

Denitrification occurs mainly in the upper portion of sediment. Therefore, porewater data (Fig 2, Table 1) were obtained from the first (0–5 cm) and second (5–10 cm) layers of sediment samples from the unplanted control and cane shoot ditches. Data from the unplanted control ditch (S1 and S2 Figs, S3 and S4) Tables were obtained from eight total samples of overlying water and sediment porewater.

**Table 1:** Analysis of the relationship between NO_3_- and the (DOC:NO_3_-) ratio in major ecosystems along the rainfall transport route.

	Modeled parameter(*y* = *ax* ^(*b*)^)		Model fit(*r* ^2^)	*N*	Gap of *Y* (range of *x*)				
	*a*	*b*			1–10	10–20	20–40	40–80	80–160
**All data**	0.87	–0.93	0.87**	411	0.77	0.049	0.025	0.013	0.0070
**Experimental systems**	0.53	–0.86	0.83**	44	0.46	0.033	0.018	0.0010	0.0055
**River**	0.26	–0.76	0.87**	34	0.21	0.019	0.011	0.0065	0.0038
**Rainfall**	0.37	–1.50	0.86*	8	0.36	0.0076	0.0027	0.00094	0.00033
**Porewater**	0.89	–0.86	0.65**	31	0.77	0.055	0.030	0.017	0.0092
**Groundwater**	0.94	–0.96	0.92**	132	0.84	0.050	0.026	0.013	0.0068
**Drainage ditch**	1.07	–0.88	0.96**	138	0.93	0.064	0.035	0.019	0.010
**Runoff**	1.70	–1.06	0.84**	24	1.55	0.077	0.037	0.018	0.0085

*N* = number of samples; Gap of *Y* = *ax*_1_^(*b*)^—*ax*_2_^(*b*)^.

* and ** indicated significant level at 0.05 and 0.01 level, respectively.

All statistical analyses were performed with SPSS 17.0. Nonlinear regression models were used to evaluate relationships between DOC:NO_3_^-^ ratios and NO_3_^-^ concentrations. Then, analysis of variance (ANOVA) was used to test the validity of these regressions. ANOVA with the least significant difference (LSD) test was applied to determine if there were statistically significant differences in the DOC concentrations, NO_3_^-^ concentrations, and DOC:NO_3_^-^ ratios between different ecosystems, between drainage ditch water at different times, and between groundwater at different times. Pearson correlation analysis was employed to test the relationship between the DOC and NO_3_^-^ concentrations in the overlying water and in the different layers of sediment porewater. Differences were defined as statistically significant when the p-value was less than 0.05.

## Results and Discussion

### Nitrate Concentrations Change in All Ecosystems

We obtained a negative power-function equation (Fig 2a) for the relationship between the NO_3_^-^ concentration and the DOC:NO_3_^-^ ratio. The correlation coefficient reached 0.87 (*n* = 411). A good fit for the equation was obtained in the seven different ecosystems (Fig 2b–2h). The regression correlation for this equation was very good, as confirmed by the ANOVA validity test (Table 1).

Previous studies have demonstrated that the C:N ratios of microbial biomass vary widely, from a minimum of 3 to a maximum of 20 [22], and that bacterial growth efficiency values range from 5% to 80% [23,24]. Consequently, ecosystems with the same DOC concentration can have different NO_3_^-^ concentrations (S5 and S6 Tables). Taylor and Townsend [19] reported an average DOC:NO_3_^-^ ratio of 3.5 at the inflection point of exponential models across 10 ecosystems (from soil to sea). Using this ratio, they established a threshold for each ecosystem. However, these inflection points cannot be obtained mathematically, as shown by our negative power-function equations. Using the mathematical analysis, we observed that the range in NO_3_^-^ concentrations was 17.26 times larger when the DOC:NO_3_^-^ ratio ranged from 1 to 10 compared to when the ratio ranged from 10 to 20. Within the range of DOC:NO_3_^-^ ratios from 1 to 10, the range of NO_3_^-^ concentrations decreased greatly as the ratio increased (Table 1).

Similar to the decline in *k* values from soil to sea [19], the *a* values in our exponential equations decreased along the water flow route (runoff > drainage ditch > groundwater > porewater > river; Table 1). This finding implies that the NO_3_^-^ concentrations of different ecosystems changed, while the DOC concentration remained constant. Thus, the DOC in the leachate of soils and streams does not appear to be prone to degradation and has limited bioavailability [16,25].

### Changes in Drainage Ditch Nitrate Concentration during Rainfall Events

**Fig 3:**
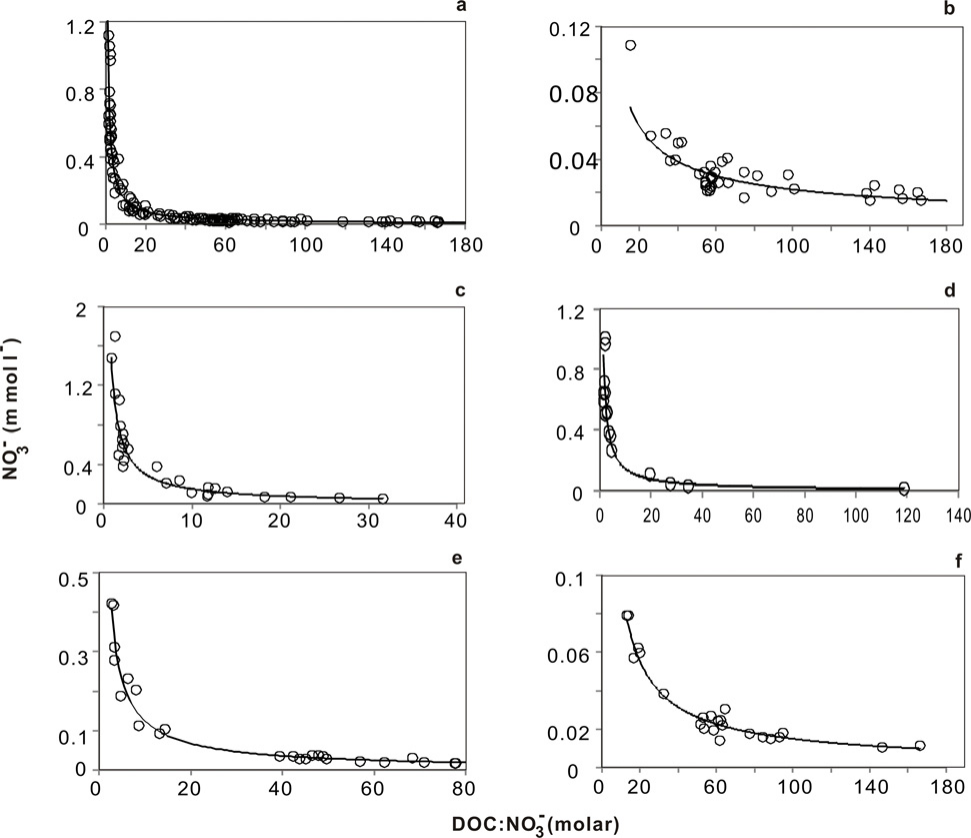
NO_3_- concentrations in the drainage ditch as a function of the molar DOC:NO_3_- ratio at different times with respect to rainfall events. a, all data; b, non-rainfall days (≥7 days after rainfall); c, final day of rainfall; d, 1 day after rainfall; e, 3 days after rainfall; f, 5 days after rainfall.

When nutrient loads from runoff flow into receiving water, their first destination is the drainage ditch. We obtained a clear power-function equation, with a high correlation coefficient, for the relationship between the NO_3_^-^ concentration in the drainage ditch and the DOC:NO_3_^-^ ratio at different times after rainfall events. This regression correlation for this equation was very good, as confirmed by the ANOVA validity test (Fig 3, Table 2).

**Table 2:** Analysis of the relationship between NO_3_- and the (DOC:NO_3_-) ratio in the drainage system according to timing with respect to rainfall events.

	Modeled parameter(*y* = *ax* ^(*b*)^)		Model fit(*r* ^2^)	N	Gap of *Y* (range of *x*)				
	*a*	*b*			1–10	10–20	20–40	40–80	80–160
**All data**	1.07	–0.88	0.96**	138	0.93	0.064	0.035	0.019	0.010
**Non-rainfall days**	0.39	–0.62	0.74**	46	0.30	0.033	0.021	0.014	0.0090
**5 days after rainfall**	0.62	–0.81	0.93**	23	0.52	0.041	0.023	0.013	0.0077
**3 days after rainfall**	0.97	–0.89	0.98**	26	0.84	0.057	0.031	0.017	0.0090
**1 day after rainfall**	1.19	–0.91	0.96**	16	1.04	0.068	0.036	0.019	0.010
**Final day of rainfall**	1.25	–0.91	0.93**	27	1.10	0.072	0.038	0.020	0.011

*N* = number of samples; Gap of *Y* = *ax*_1_^(*b*)^—*ax*_2_^(*b*)^.

** indicated significant level at 0.01.

Anabolic uptake and denitrification are two general pathways by which NO_3_^-^ accumulation is reduced. Photosynthetic reactions by phytoplankton (autotrophic uptake) occur simultaneously with bacterial growth (heterotrophic uptake) in the overlying water because the phytoplankton supply the biodegradable DOC that bacteria require for growth [23,24]. The overlying water immediately takes up NO_3_^-^ when runoff flows into the drainage ditch during a rainfall event. A similar result was found in research on Sugar Creek in central Indiana, USA [26]. In the present study, biodegradable DOC was utilized by bacteria for growth before being utilized for autotrophic uptake [27]. As a result, the DOC concentration decreased from 2.19 mmol l^-1^ before to 1.48 mmol l^-1^ after a rainfall event (S6 Table). Assuming a microbial growth efficiency of 50% and a microbial C:N ratio of 7:1 [28], we determined that 0.35 mmol C l^-1^ and 0.05 mmol N l^-1^ could be assimilated into the microbial biomass, accounting for a NO_3_^-^ concentration loss of only 10% during the entire rainfall event. This proportion of NO_3_^-^ lost is small, given that the biodegradable DOC concentration only decreased by 32.42% during rainfall events. The remaining DOC concentration (67.58%) remained constant and represented the non-biodegradable fraction (S6 Table). Sovak [29] revealed similar results in stream surface water.

Microbial reproduction should not be large [25] in an environment of water retention [28] and limited DOC bioavailability [16,30]. Accordingly, the DOC concentration did not drop significantly during 5 days after a rainfall event (S6 Table). Therefore, the decreased NO_3_^-^ concentration several days after a rainfall event depends mainly on sediment denitrification. This fact was also demonstrated by the differences in the DOC and NO_3_^-^ concentrations between the overlying water and different layers of sediment porewater (S1 and S2 Figs; S3 and S4 Tables). The observed decline in NO_3_^-^ concentration and increase in DOC concentration in the overlying water after a rainfall event can be explained by the role of the sediment as an important source of DOC [29,31]. Changes in the NO_3_^-^ concentration cannot be explained by the DOC:NO_3_^-^ ratio in the overlying water alone (S6 Table). However, the *a* values in the power-function equation and the NO_3_^-^ concentration exhibited regularly decreasing trends after rainfall (Table 2), primarily due to the disparity in DOC bioavailability caused by differences in supply volume and velocity [16,25].

Taken together, these results indicate that the overlying water and sediment are inseparable parts of aquatic ecosystems. Sediment status and depth must be considered in the creation of artificial aquatic ecosystems (e.g., drainage ditches, reservoirs, canals, and aquaculture ponds). In contrast, measures for sediment removal have no benefit in terms of N removal.

### Changes in Groundwater Nitrate Concentration during Rainfall Events

**Fig 4:**
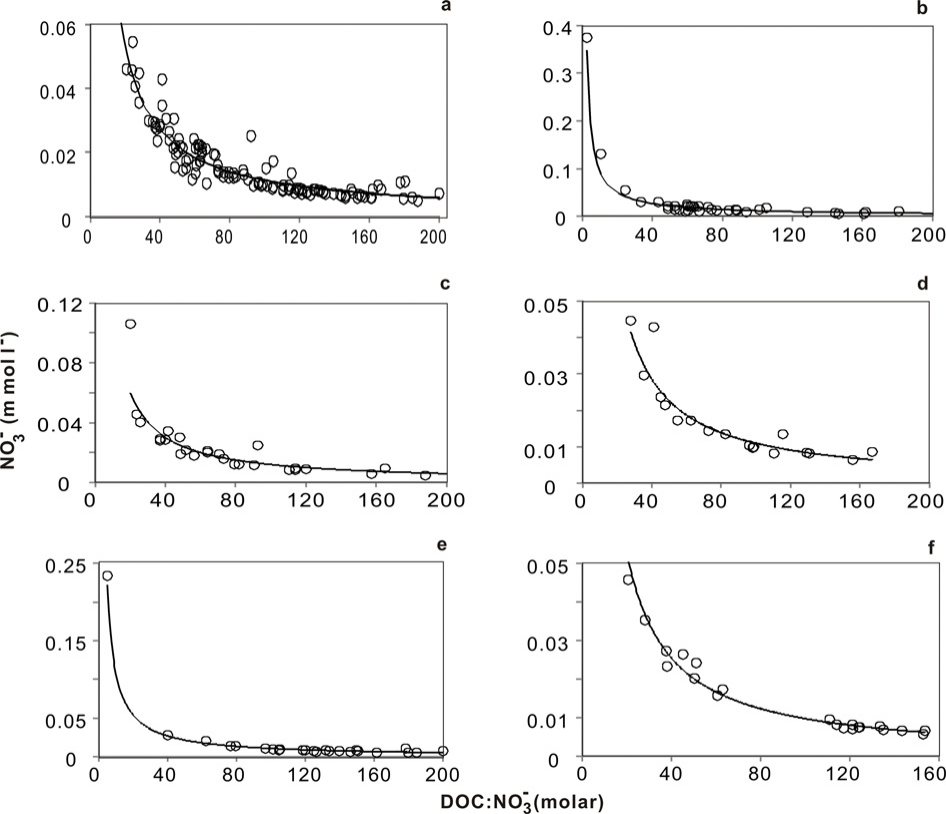
NO_3_- concentrations in groundwater as a function of the molar DOC:NO_3_- ratio at different times with respect to rainfall events. a, all data; b, non-rainfall days (≥7 days after rainfall); c, final day of rainfall; d, 1 day after rainfall; e, 3 days after rainfall; f, 5 days after rainfall.

We obtained a clear power-function equation, with a high correlation coefficient, for the relationship between the NO_3_^-^ concentration in groundwater and the DOC:NO_3_^-^ ratio at different times with respect to rainfall events. The regression correlation for this equation was very good, as confirmed by the validity test (Fig 4, Table 3). Most NO_3_^-^ concentrations were less than 1 mmol l^-1^. The DOC:NO_3_^-^ ratios exceeded 20 (Fig 4), higher than the ratio obtained in wells with large NO_3_^-^ accumulation (<10) [32,33]. Low DOC:NO_3_^-^ ratios can exist only when low DOC concentrations induce NO_3_^-^ accumulation in the environment by restricting denitrification [34,35]. The addition of exogenous DOC is necessary at many groundwater sites to sustain low NO_3_^-^ concentrations [31,36]. Soil leaching induced only a slight increase in NO_3_^-^ concentration 1 day after a rainfall event, mainly due to dilution by a large groundwater pool (S7 Table). High DOC concentrations in groundwater were observed at different times after rainfall events (S7 Table) and in different agricultural ecosystems (S5 Table). These observations indicate that the addition of exogenous organic material is one important way to sustain low NO_3_^-^ concentrations in manure-fertilized farmland. Taken together, these results indicate that continuous application of high-N, low-C fertilizer may increase the threat of NO_3_^-^ accumulation in farmland groundwater.

**Table 3:** Analysis of the relationship between NO_3_-and the (DOC:NO_3_-) ratio in groundwater system according to timing with respect to rainfall events.

	Modeled parameter(*y* = *ax* ^(*b*)^)		Model fit(*r* ^2^)	*N*	Gap of *Y* (range of *x*)				
	*a*	*b*			1–10	10–20	20–40	40–80	80–160
**All data**	0.94	–0.96	0.92**	132	0.84	0.050	0.026	0.013	0.0068
**Non-rainfall days**	0.74	–0.90	0.92**	39	0.65	0.043	0.023	0.012	0.0066
**5 days after rainfall**	1.18	–1.04	0.98**	21	1.07	0.055	0.027	0.013	0.0064
**3 days after rainfall**	1.06	–1.00	0.95**	24	0.95	0.053	0.026	0.013	0.0066
**1 day after rainfall**	1.31	–1.04	0.90**	18	1.19	0.061	0.030	0.014	0.0071
**Final day of rainfall**	1.21	–1.00	0.90**	30	1.09	0.060	0.030	0.015	0.0076

*N* = number of samples; Gap of *Y* = *ax*_1_^(*b*)^—*ax*_2_^(*b*)^.

** indicated significant level at 0.01.

## Supporting Information

S1 Fig Dissolved organic carbon (DOC) concentrations in overlying water (OW) and different layers of sediment porewater (PW). (TIF)

S2 Fig Nitrate (NO_3_-) concentrations in overlying water (OW) and different layers of sediment porewater (PW). (TIF)

S1 Table Details of experimental ecosystems. (DOCX)

S2 Table Details of sampling. (DOCX)

S3 Table Pearson correlation analysis of DOC concentrations in overlying water (OW) and different layers of sediment porewater (PW) (*n* = 8). (DOCX)

S4 Table Pearson correlation analysis of NO_3_- concentrations in overlying water (OW) and different layers of sediment porewater (PW) (*n* = 8). (DOCX)

S5 Table Analysis of variance in the DOC and NO_3_- concentrations (mmol l^-1^) and the DOC:NO_3_- ratios in different ecosystems. (DOCX)

S6 Table Analysis of variance in the DOC and NO_3_- concentrations (mmol l^-1^) and the DOC:NO_3_- ratios in drainage ditch water according to the timing of rainfall events. (DOCX)

S7 Table Analysis of variance in the DOC and NO_3_- concentrations (mmol l^-1^) and the DOC:NO_3_- ratios in groundwater according to the timing of rainfall events. (DOCX)
