# Supplementary figures and images for: Stoichiometric Determination of Nitrate Fate in Agricultural Ecosystems during Rainfall Events
Source: PLoS One. 2015 Apr 7;10(4):e0122484. doi: 10.1371/journal.pone.0122484 (PMC4388451; doi:10.1371/journal.pone.0122484)

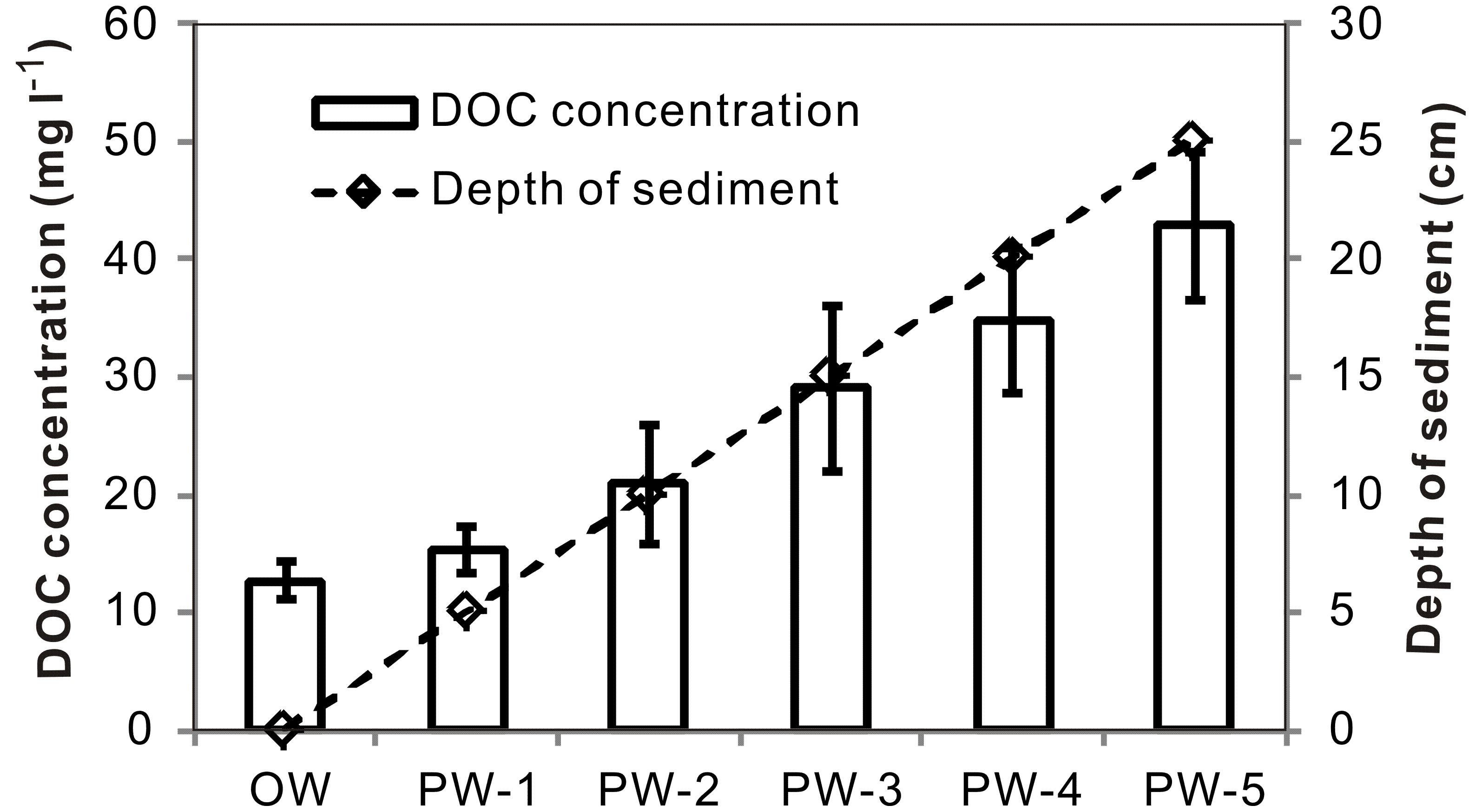

Supplement: S1 Fig — (TIF) [file pone.0122484.s001.tif]

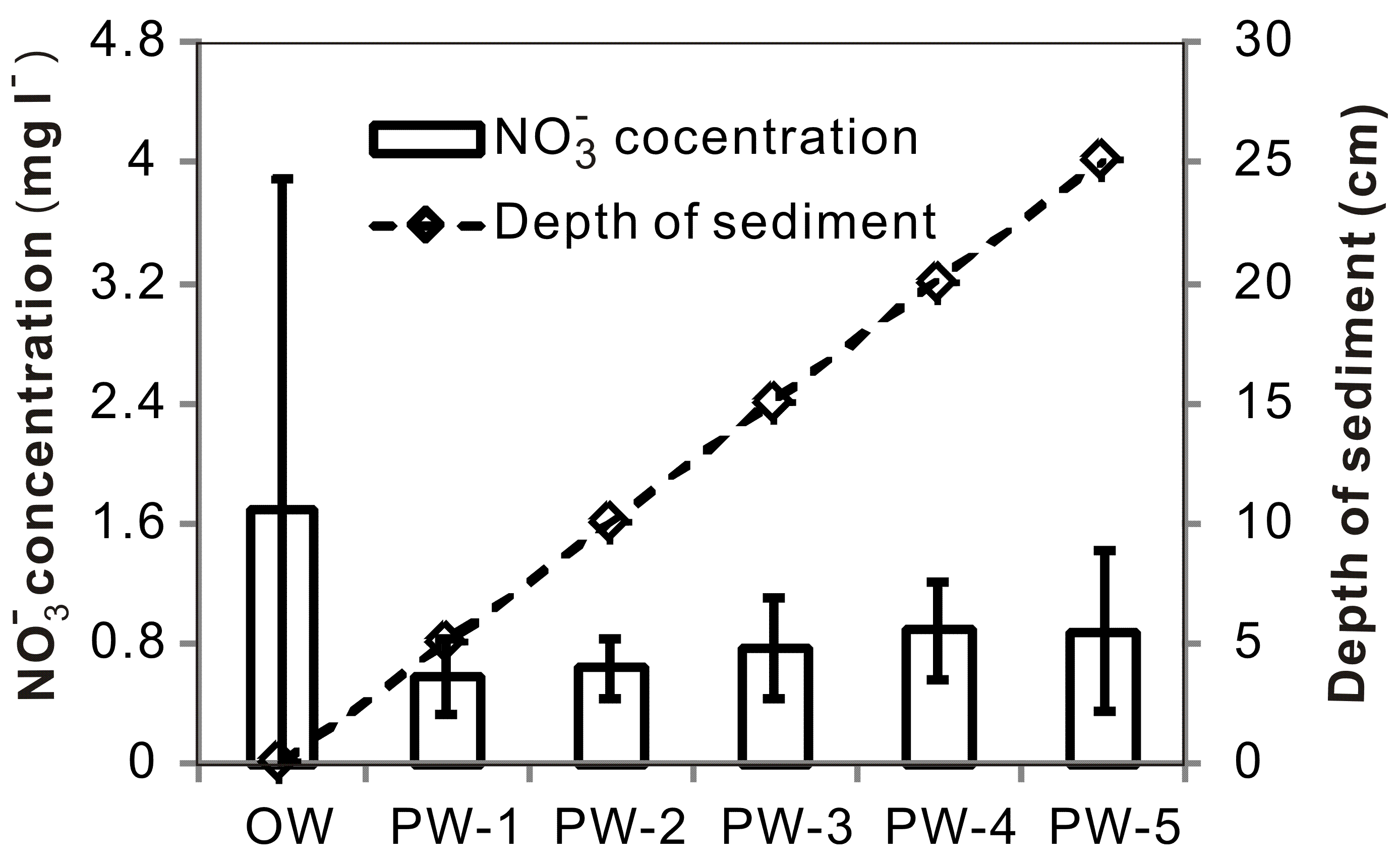

Supplement: S2 Fig — (TIF) [file pone.0122484.s002.tif]
