# Supplementary material for: Stoichiometric Determination of Nitrate Fate in Agricultural Ecosystems during Rainfall Events
Source: PLoS One. 2015 Apr 7;10(4):e0122484. doi: 10.1371/journal.pone.0122484 (PMC4388451; doi:10.1371/journal.pone.0122484)
Supplement: S1 Table — (DOCX) [file pone.0122484.s003.docx]

**S1 Table:** Details of experimental ecosystems.

| System | Tank volume (L) | Sediment source | Sediment volume (L) | Original water source | Original water volume (L) | Duckweed (g) | Runoff  volume (L) |
| --- | --- | --- | --- | --- | --- | --- | --- |
| 1 | 40 | Drainage ditch | 10 | - | - | - | 10 |
| 2 | 40 | Lake | 10 | - | - | - | 10 |
| 3 | 40 | Drainage ditch | 10 | Drainage ditch | 10 | - | 10 |
| 4 | 40 | Drainage ditch | 10 | Drainage ditch | 10 | 40 | 10 |
| 5 | 100 | Drainage ditch | 25 | - | - | - | 25 |
| 6 | 100 | Drainage ditch | 25 | Drainage ditch | 25 | - | - |
| 7 | 100 | Lake | 25 | - | - | - | 25 |

- = no input.
