# Supplementary material for: Stoichiometric Determination of Nitrate Fate in Agricultural Ecosystems during Rainfall Events
Source: PLoS One. 2015 Apr 7;10(4):e0122484. doi: 10.1371/journal.pone.0122484 (PMC4388451; doi:10.1371/journal.pone.0122484)
Supplement: S2 Table — (DOCX) [file pone.0122484.s004.docx]

**S2 Table:** Details of sampling.

| Date(2013) | Weather | Rainfall | Runoff | Groundwater | Drainage ditch | River | Porewater | Experimental ecosystem |
| --- | --- | --- | --- | --- | --- | --- | --- | --- |
| 22 Apr | N | - | - | 3 | 2(UC, DW) | 1 (SR) | - | - |
| 23 Apr | 0(13.5mm) | 1 | 3 | 3 | 2(UC, DW) | 1 (SR) | - | - |
| 24 Apr | 1 | - | - | 3 | 2(UC, DW) | 1 (SR) | - | - |
| 26 Apr | 3 | - | - | 3 | 2(UC, DW) | 1 (SR) | - | - |
| 7 May | N | - | - | 3 | - | - | - | - |
| 10 May | 0(15.8mm) | 1 | - | 3 | - | - | - | - |
| 11 May | 1 | - | - | 3 | - | - | - | - |
| 13 May | 3 | - | - | 3 | - | - | - | - |
| 16 May | N | - | - | 3 | 4 | 1 (SR) | - | - |
| 17 May | 0(30.6mm) | 1 | 3 | 3 | 4 | 1 (SR) | - | - |
| 18 May | 1 | - | - | 3 | 4 | 1 (SR) | - | - |
| 20 May | 3 | - | - | 3 | 4 | 1 (SR) | - | - |
| 22 May | 5 | - | - | 3 | 4 | 1 (SR) | - | - |
| 1 Jun | 0(36.1mm) | 1 | 3 | 3 | 4 | 1 (SR) | - | - |
| 2 Jun | 1 | - | - | 3 | 4 | 1 (SR) | - | - |
| 4 Jun | 3 | - | - | 3 | 4 | 1 (SR) | - | - |
| 6 Jun | 5 | - | - | 3 |  |  | - | - |
| 8 Jun | 0(90.5mm) | 1 | 3 | 3 | 4 | 1 (LR) | - | - |
| 9 Jun | 1 | - | - | 3 | 4 | 1 (LR) | - | - |
| 11 Jun | 3 | - | - | 3 | 4 | 1 (LR) | - | - |
| 13 Jun | 5 | - | - | 3 | 4 | 1 (LR) | - | - |
| 26 Jun | 0(26.4mm) | 1 | 3 | 3 | 4 | 1 (LR) | - | - |
| 27 Jun | 0(9.3mm) | 1 | 3 | 3 | 4 | 1 (LR) | - | - |
| 28 Jun | 1 | - | - | 3 | 4 | 1 (LR) | - | - |
| 30 Jun | 3 | - | - | 3 | 4 | 1 (LR) | - | - |
| 2 Jul | 5 | - | - | 3 | 4 | 1 (LR) | - | - |
| 7 Jul | 0(59.3mm) | 1 | 3 | 3 | 4 | 1 (LR) | - | - |
| 9 Jul | 1 | - | - | 3 | 4 | 1 (LR) | - | - |
| 12 Jul | 5 | - | - | 3 | 4 | 1 (LR) | - | - |
| 18 Jul | N | - | - | 3 | 4 | 1 (LR) | - | - |
| 21 Jul | N | - | - | 3 | 4 | 1 (LR) | - | - |
| 23 Jul | N | - | - | 3 | 4 | 1 (LR) | - | - |
| 25 Jul | N | - | - | 3 | 4 | 1 (LR) | - | - |
| 28 Jul | N | - | - | 3 | 4 | 1 (LR) | - | - |
| 30 Jul | N | - | - | 3 | 4 | 1 (LR) | - | - |
| 1 Aug | N | - | - | 3 | 4 | 1 (LR) | - | - |
| 2 Aug | 0(10.6mm) | - | - | 3 | 4 | 1 (LR) | - | - |
| 6 Aug | 3 | - | - | 3 | 4 | 1 (LR) | - | - |
| 8 Aug | 5 | - | - | 3 | - | 1 (LR) | - | - |
| 13 Aug | N | - | - | 3 | - | - | - | - |
| 15 Aug | 0 | - | - | - | - | - | - | 4 (S) |
| 16 Aug | 1 | - | - | - | - | - | - | 4 (S) |
| 18 Aug | 3 | - | - | 3 | - | - | - | 4 (S) |
| 20 Aug | 5 | - | - |  | - | - | - | 4 (S) |
| 26 Aug | 0(45mm) | - | 3 | 3 | 4 | - | 5 (BC) | - |
| 29 Aug | 3 | - | - | 3 | 4 | - | 5 (BC) | - |
| 31 Aug | 5 | - | - | 3 | 4 | - | 5 (BC) | - |
| 5 Sep | 0 | - | - | - | - | - | - | 7 |
| 6 Sep | 1 | - | - | - | - | - | - | 7 |
| 8 Sep | 3 | - | - | - | - | - | - | 7 |
| 10 Sep | 5 | - | - | - | - | - | - | 7 |
| 13 Sep | N | - | - | - | - | - | 10 | - |
| 15 Sep | 0 | - | - | - | - | - | 10 | - |
| 18 Sep | 3 | - | - | - | - | - | 10 | - |
| 20 Sep | 5 | - | - | - | - | - | 10 | - |
| 22 Sep | 7 | - | - | - | - | - | 10 | - |

In the weather column, precipitation amounts are reported in millimeters; 0, 1, 3, 5, and 7 denote the number of days after rainfall; N = non-rainfall day. In other columns, numerals indicate number of samples; - = no sampling. UC = unplanted control; DW = duckweed; SR = small river; LR = large river; S = small.
