# Supplementary material for: Stoichiometric Determination of Nitrate Fate in Agricultural Ecosystems during Rainfall Events
Source: PLoS One. 2015 Apr 7;10(4):e0122484. doi: 10.1371/journal.pone.0122484 (PMC4388451; doi:10.1371/journal.pone.0122484)
Supplement: S3 Table — (DOCX) [file pone.0122484.s005.docx]

**S3 Table:** Pearson correlation analysis of DOC concentrations in overlying water (OW) and different layers of sediment porewater (PW) (*n* = 8)

|  | PW-1 | PW-2 | PW-3 | PW-4 | PW-5 |
| --- | --- | --- | --- | --- | --- |
| OW | 0.88** | 0.68 | 0.73* | 0.56 | 0.46 |
| PW-1 |  | 0.84** | 0.88** | 0.71* | 0.70 |
| PW-2 |  |  | 0.99** | 0.95** | 0.86** |
| PW-3 |  |  |  | 0.95** | 0.90** |
| PW-4 |  |  |  |  | 0.93** |

PW-1 = 0–5 cm; PW-2 = 5–10 cm; PW-3 = 10–15 cm; PW-4 = 15–20 cm; PW-5 = 20–25 cm. **p* ≤ 0.05; ***p* ≤ 0.01.
