# Supplementary material for: Stoichiometric Determination of Nitrate Fate in Agricultural Ecosystems during Rainfall Events
Source: PLoS One. 2015 Apr 7;10(4):e0122484. doi: 10.1371/journal.pone.0122484 (PMC4388451; doi:10.1371/journal.pone.0122484)
Supplement: S5 Table — (DOCX) [file pone.0122484.s007.docx]

**S5 Table:** Analysis of variance in the DOC and NO_3_^-^ concentrations (mmol l^-1^) and the DOC:NO_3_^-^ ratios in different ecosystems.

|  | Rainfall | Runoff | Drainage ditch | Groundwater | Porewater | River |
| --- | --- | --- | --- | --- | --- | --- |
| DOC | 0.20 ± 0.14^a^ | 1.87 ± 1.17^d^ | 1.68 ± 0.73^cd^ | 1.15 ± 0.29^b^ | 1.50 ± 0.28^c^ | 0.56 ± 0.33^a^ |
| NO_3_^-^ | 0.027 ± 0.016^ab^ | 1.18 ± 1.09^c^ | 0.18 ± 0.30^b^ | 0.021 ± 0.040^a^ | 0.042 ± 0.013^a^ | 0.042 ± 0.033^a^ |
| DOC:NO_3_^-^ | 8.12 ± 4.75^ab^ | 4.21 ± 3.70^a^ | 57.54 ± 68.01^b^ | 108.62 ± 97.59^c^ | 37.77 ± 9.45^a^ | 35.27 ± 61.93^a^ |

Data are presented as means ± standard deviations. Superscripted letters in rows indicate significant differences (*p* < 0.05).
