# Supplementary material for: Stoichiometric Determination of Nitrate Fate in Agricultural Ecosystems during Rainfall Events
Source: PLoS One. 2015 Apr 7;10(4):e0122484. doi: 10.1371/journal.pone.0122484 (PMC4388451; doi:10.1371/journal.pone.0122484)
Supplement: S6 Table — (DOCX) [file pone.0122484.s008.docx]

**S6 Table:** Analysis of variance in the DOC and NO_3_^-^ concentrations (mmol l^-1^) and the DOC:NO_3_^-^ ratios in drainage ditch water according to the timing of rainfall events.

|  | BR | AR-0 | AR-1 | AR-3 | AR-5 |
| --- | --- | --- | --- | --- | --- |
| DOC | 2.19 ± 0.98^a^ | 1.48 ± 0.42^b^ | 1.43 ± 0.41^b^ | 1.42 ± 0.32^b^ | 1.35 ± 0.28^b^ |
| NO_3_^-^ | 0.028 ± 0.017^a^ | 0.48 ± 0.44^b^ | 0.46 ± 0.31^b^ | 0.11 ± 0.13^ac^ | 0.031 ± 0.021^ac^ |
| DOC:NO_3_^-^ | 104.87 ± 84.13^a^ | 8.04 ± 8.36^b^ | 14.42 ± 29.71^bc^ | 46.80 ± 50.74^cd^ | 63.12 ± 38.76^de^ |

Data are presented as means ± standard deviations. Superscripted letters in rows indicate significant differences (*p* < 0.05). BR = before rainfall; AR = after rainfall; 0, 1, 3, and 5 denote number of days after rainfall.
