# Supplementary material for: Stoichiometric Determination of Nitrate Fate in Agricultural Ecosystems during Rainfall Events
Source: PLoS One. 2015 Apr 7;10(4):e0122484. doi: 10.1371/journal.pone.0122484 (PMC4388451; doi:10.1371/journal.pone.0122484)
Supplement: S7 Table — (DOCX) [file pone.0122484.s009.docx]

**S7 Table:** Analysis of variance in the DOC and NO_3_^-^ concentrations (mmol l^-1^) and the DOC:NO_3_^-^ ratios in groundwater according to the timing of rainfall events.

|  | BR | AR-0 | AR-1 | AR-3 | AR-5 |
| --- | --- | --- | --- | --- | --- |
| DOC | 1.20 ± 0.36^a^ | 1.25 ± 0.33^a^ | 1.13 ± 0.23^ab^ | 1.10 ± 0.21^ab^ | 1.00 ± 0.097^b^ |
| NO_3_^-^ | 0.027 ± 0.061^a^ | 0.020 ±0.019^a^ | 0.017 ± 0.011^a^ | 0.019 ± 0.046^a^ | 0.016 ± 0.011^a^ |
| DOC:NO_3_^-^ | 129.38 ± 160.12^a^ | 95.26 ±62.17^a^ | 87.33 ± 41.93^a^ | 121.44 ± 46.78^a^ | 92.77 ± 45.35^a^ |

Data are presented as means ± standard deviations. Superscripted letters in rows indicate significant differences (*p* < 0.05). BR = before rainfall; AR = after rainfall; 0, 1, 3, and 5 denote number of days after rainfall.
